# Mirror-Peptidizer: In Silico Mirror-Image Screening Enables De Novo Design of D-Peptide Binders without D-Protein Synthesis

**DOI:** 10.34133/research.1420

**Published:** 2026-08-31

**Authors:** Bohan Ma, Zhe Wang, Yanlin Jian, Yonghong Mi, Si Chen, Honggang Hu, Xiang Li

**Affiliations:** ^1^ Department of Urology, The First Affiliated Hospital, Xi’an Jiaotong University, Xi’an 710049, China.; ^2^ Institute of Bioengineering, College of Chemical and Biological Engineering, Zhejiang University, Hangzhou 310000, China.; ^3^ School of Pharmacy, Second Military Medical University, Shanghai 200433, China.; ^4^ School of Medicine, Shanghai University, Shanghai 200444, China.

## Abstract

D-peptides are attractive therapeutic modalities because they are generally more resistant to proteolysis than their L-counterparts, yet systematic design of target-binding D-peptides remains nontrivial. Here, we report Mirror-Peptidizer, an end-to-end in silico mirror-image screening workflow that generates D-peptide binders without requiring chemical synthesis of D-protein targets. The workflow mirrors an L-protein structure to a virtual D-protein, designs L-peptide backbones in the presence of the mirrored target using a diffusion-based backbone generator, selects sequences with a neural sequence design model, and explores local sequence neighborhoods via Bayesian multi-objective optimization balancing sequence-backbone compatibility and a solubility heuristic. Mirroring the resulting complex yields the corresponding D-peptide predicted to bind the native L-target. Using MDM2, PD-L1, and interleukin-23 receptor (IL-23R) as test cases, we identified D-peptides spanning α-helical, β-rich, and mixed conformations with affinity from 11.9 nM to sub-μM. For the MDM2 system, the ^1^H-^15^N HSQC (heteronuclear single quantum coherence) perturbations and protein mutagenesis support the designed interface, and cell-penetrating conjugates show p53-dependent cancer growth inhibition. Similarly, PD-L1-targeting D-peptides potently inhibited PD-1/PD-L1 interactions in competitive binding assays in vitro*,* and IL-23R-targeting D-peptides inhibited IL-2/IL-12/IL-23-induced interferon-γ production in human peripheral blood mononuclear cells. Mirror-Peptidizer is provided as an open-source implementation to facilitate rapid generation of experimentally testable D-peptide starting points.

## Introduction

D-peptides, composed of D-amino acids, are enantiomeric counterparts of naturally occurring L-peptides and are widely studied as ligands and inhibitors because D-configurations are generally less susceptible to proteases and can exhibit favorable stability profiles [1,2]. Consequently, D-peptides represent a promising class of therapeutic agents, particularly well suitable for membrane proteins and secreted signaling molecules, including PD-1/PD-L1 immune checkpoint pathway [3] and the interleukin-23 receptor (IL-23R) [4]. Mirror-image phage display provides a powerful route to discover D-peptide ligands by screening L-peptides against a chemically synthesized D-protein target, followed by enantiomeric conversion of the selected peptide [5,6]. However, the requirement for preparing folded D-proteins and performing experimental display selections limits throughput and general applicability, especially for larger or complex targets.

The structural prediction and design of D-peptides remain a formidable challenge in computational biology [7,8]. Despite the advancements of AlphaFold 3 in predicting protein–ligand interactions and multi-molecular complexes with up to 76% accuracy for certain targets, its performance in D-peptide modeling reveals critical shortcomings [9]. Recent analyses indicate that AlphaFold 3 exhibits an average chiral error rate of 51% for D-peptide structures, a result indistinguishable from random guessing. Several computational approaches have been reported to support D-peptide discovery [10], including structure-based identification tools [11] and α-helix hot spot identification [12]. In parallel, recent work has demonstrated that coordinate mirroring combined with modern design algorithms can produce binders in opposite chirality, often coupled to high-throughput experimental testing and affinity maturation [13].

These developments motivate a practical question: Can an integrated, fully computational workflow generate experimentally testable D-peptide binders directly from an L-target structure, without D-protein synthesis and without display-based affinity maturation? Here, we present Mirror-Peptidizer, a pragmatic in silico mirror-image screening pipeline that integrates coordinate mirroring, diffusion-based binder backbone generation, neural sequence design, and Bayesian multi-objective sequence exploration. Instead of directly generating a D-peptide structure, our approach begins by fixing the target protein to its corresponding D-protein counterpart. L-peptide is then generated through established screening methods, and the resulting structure is computationally mirrored to yield the desired D-peptide. To further enhance the process, we coupled in silico mirroring with Bayesian optimization (BO) [14,15]—a probabilistic method that efficiently explores complex parameter spaces. This integration allowed us to develop a computational pipeline for de novo D-peptide drug generation, addressing both data limitations and the stereochemical challenges inherent to D-peptide design. Overall, this innovative approach leverages established design techniques while incorporating advanced computational methods, potentially paving the way for more robust D-peptide therapeutics.

To demonstrate the robustness of Mirror-Peptidizer, we applied it to 3 therapeutically relevant targets: MDM2 [16], PD-L1 [17], and IL-23R [18]. These proteins play critical roles in cancer and immune diseases, making them high-priority candidates for drug development. Using our method, we successfully designed D-peptides with low nanomolar binding affinities for each target. A series of wet-experiments, including isothermal titration calorimetry (ITC) binding assay of wild-type protein and site-directed mutagenesis, nuclear magnetic resonance (NMR) spectrum of D-peptide complexed with MDM2, confirmed the high specificity and accurate binding mode of these peptides. Notably, the D-peptides targeting MDM2 were conjugated with a cell-penetrating peptide to enhance intracellular delivery. These conjugates effectively entered cancer cells and induced p53-dependent apoptosis, highlighting their potential as a novel therapeutic strategy for cancer treatment. The universal nature of Mirror-Peptidizer establishes a mirror-coordinate design strategy in peptide drug discovery, enabling the rapid and precise design of D-peptides for diverse therapeutic targets, addressing longstanding challenges in mirror-image peptide design, and opening new avenues for tackling complex diseases.

## Results

### Mirror-Peptidizer for designing mirror-peptide binders

**Fig. 1.**
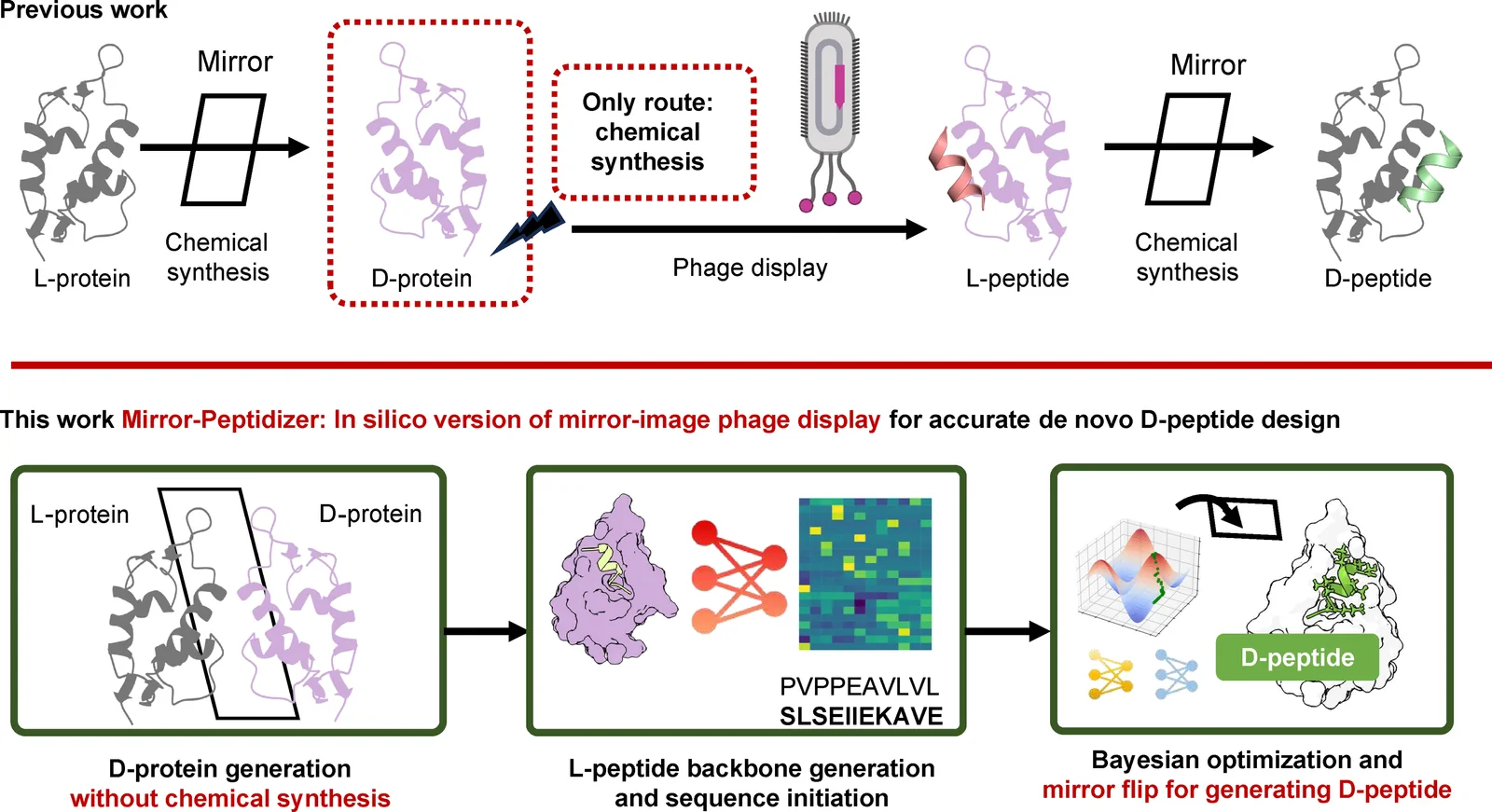
Comparison of the mirror-image phage display technique and the Mirror-Peptidizer pipeline for mirror-peptide design. The mirror-image phage display technique requires the chemical synthesis of a D-protein, whereas the Mirror-Peptidizer pipeline is a fully computational, step-by-step approach. First, the target L-protein structure is computationally mirrored to generate a virtual D-protein. Next, in silico design was used to generate an L-peptide that binds to the virtual D-protein. Finally, the resulting L-protein/D-peptide complex is optimized for the final D-peptide design, which is ready for experimental validation. This pipeline provides an accurate and efficient solution for mirror-peptide design.

In previous studies, the most well-established method for designing D-peptides has been the mirror-image phage display technique [19]. This approach involves synthesizing the D-protein corresponding to the target L-protein, followed by phage display screening to identify an L-peptide that binds to the synthesized D-protein [20]. The identified L-peptide is then “flipped” to generate the corresponding D-peptide (Fig. 1). While effective in certain cases, this method is highly labor-intensive, requiring extensive protein synthesis and screening efforts. Moreover, the process suffers from a low success rate, making it inefficient and unreliable for broader applications. To address these limitations, we developed a novel pipeline called Mirror-Peptidizer, which eliminates the need for labor-intensive D-protein synthesis and simplifies the D-peptide design process (Fig. 1).

The Mirror-Peptidizer workflow begins by generating a mirror-image target protein. Specifically, the native L-configured target protein is chirality-inverted by applying a stereochemical coordinate transformation to the target PDB coordinates along the *x* axis, although the *y* or *z* axis can be used mathematical equivalently. This step is crucial to ensure that the inverted protein retains its structural integrity while being optimally configured for D-peptide binding. Following this, an L-peptide backbone is generated to bind the fixed D-form target by Chroma. Chroma plays a central role in the second step of the pipeline, where it is responsible for generating the spatial backbone conformation of the L-peptide complexed with the target D-protein [21]. The core of Chroma’s functionality is a conditional generative diffusion model powered by an E(3)-equivariant graph neural network (GNN). This architecture enables the model to accurately represent and manipulate 3-dimensional molecular geometries. In this step, Chroma produces a preliminary 3-dimensional backbone structure that conforms to the binding surface of the D-protein target. This generated backbone serves as the structural foundation for the subsequent stage of sequence design, where side chains and amino acid identities are optimized to achieve high-affinity binding and stability. After generating the peptide backbone, we employed the deep learning framework ProteinMPNN to design and optimize the side chain sequences and conformations, thereby refining the peptide to achieve favorable binding affinity and structural stability [22]. ProteinMPNN was employed as the sequence-design engine following Chroma backbone generation. Rather than designing sequences directly for D-peptides, Mirror-Peptidizer performs sequence assignment in a mirrored L-coordinate system, where standard L-amino-acid residue identities can be predicted using the pretrained ProteinMPNN model. During sequence design, receptor chains were kept fixed, while only the peptide chain was redesigned, allowing the generated sequences to adapt to the local receptor geometry. ProteinMPNN evaluates sequence-backbone compatibility through residue-level conditional probabilities derived from the surrounding 3-dimensional environment, thereby favoring amino-acid combinations that are structurally consistent with the designed peptide backbone.

**Fig. 2.**
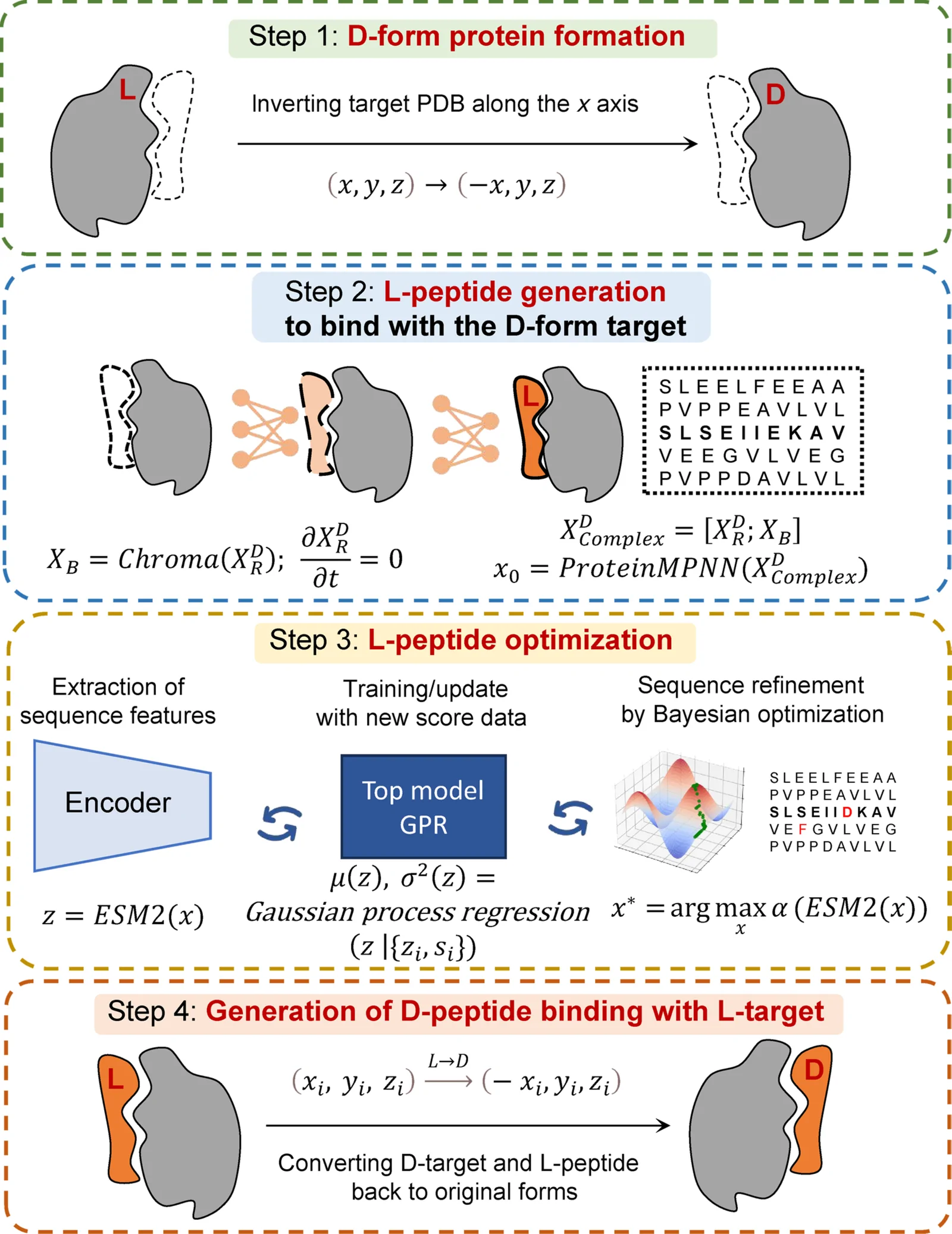
The workflow of the Mirror-Peptidizer algorithm for de novo D-peptide design. This schematic outlines the computational pipeline integrating in silico mirroring and BO to generate enantiomeric D-peptide therapeutics.

These predicted structures are then subjected to a multi-parameter BO process that iteratively refines the peptide sequences [23]. This process uses BO to iteratively improve the peptide sequences, aiming to enhance sequence-backbone compatibility while considering the solubility-weighted index (SWI). In our pipeline, multi-objective optimization is employed to balance sequence-backbone compatibility, structural stability, and peptide solubility, thereby avoiding the generation of candidates with poor physicochemical properties. Rather than using a simple weighted-sum approach, we adopt a desirability-based scoring framework. The advantage of this multi-objective optimization algorithm is that the contribution of critical objectives is not diluted as additional factors are introduced, effectively preventing the selection of peptide candidates with severe deficiencies in key properties. Following BO, ProteinMPNN is used again to score the mutated sequences, guiding the search toward candidates with improved structural compatibility. To integrate the ProteinMPNN score and the SWI, we employed importance-weighted desirability scoring to generate an overall FuzzyScore. If any individual score is very poor, the overall FuzzyScore approaches zero, effectively filtering out candidates with critical deficiencies. Mirror-Peptidizer ranks candidates using a design-prioritization score rather than a direct binding-affinity prediction. The workflow does not calculate binding free energy, docking score, or physics-based interaction energy for the final heterochiral L-protein/D-peptide complex. Instead, ProteinMPNN score is used as a lower-is-better sequence–backbone compatibility metric, whereas the SWI/synthesis-related term penalizes candidates with poor solubility or synthetic tractability. FuzzyScore integrates these normalized desirability values to prioritize candidates that are structurally plausible and experimentally tractable. Thus, computational ranking was used to select candidates for synthesis and validation, while binding affinity was determined experimentally by ITC and related assays (Fig. 2).

By combining virtual protein flipping, advanced diffusion model algorithms, and sequence optimization tools, our streamlined and efficient method represents an important advancement over traditional mirror-image phage screening, providing a candidate-generation workflow to longstanding challenges in D-peptide development. The full implementation of the Mirror-Peptidizer pipeline has been open-sourced and is freely accessible at https://github.com/dahuilangda/Mirror-Peptidizer.

### The design of MDM2-binding D-peptides through Mirror-Peptidizer

**Fig. 3.**
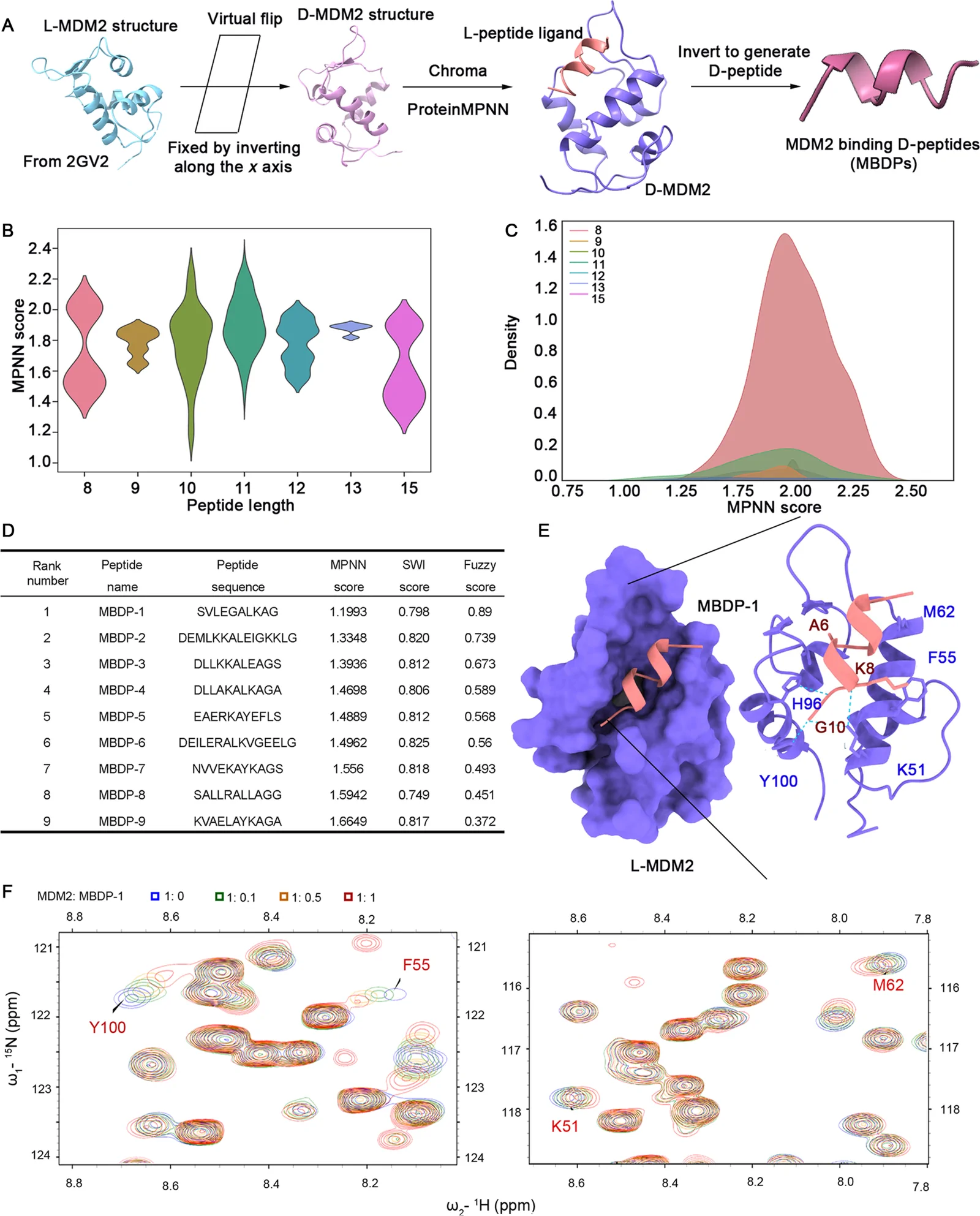
Computational design of D-peptides targeting MDM2. (A) Schematic overview of the Mirror-Peptidizer pipeline for designing MDM2-binding D-peptides (MBDPs). (B) Plot of MBDP length versus MPNN design score. (C) Density plots illustrating the distribution of MBDP lengths and MPNN scores. (D) Top 9 MBDP sequences and their corresponding MPNN scores. (E) Detailed structural analysis of MBDP1 binding to MDM2 protein. (F) Selected ^1^H−^15^N HSQC spectra of MDM2, obtained during the automated titration experiment with MBDP-1.

To validate Mirror-Peptidizer, we selected MDM2 as a representative target protein due to its critical role in cancer biology and its well-characterized interactions with peptide ligands. Using the algorithm, we began by virtually mapping the L-protein structure of MDM2 (PDB: 2GV2) [24] to generate its corresponding D-protein structure through a coordinate inversion along the *x* axis. Subsequently, we applied our algorithm to design an L-peptide targeting this virtual D-protein. The designed L-peptide was then flipped to produce the D-peptide structure, yielding MDM2-binding D-peptides, termed MBDPs (Fig. 3A).

The algorithm demonstrated consistent performance across peptides of varying lengths, as reflected by the relatively uniform MPNN evaluation scores (Fig. 3B). This highlights the robustness of the sequence optimization process implemented by ProteinMPNN. Additionally, MBDPs of length 10 exhibited the highest density of optimal scores, indicating that this length is particularly suitable for effective binding to MDM2 (Fig. 3C). Several peptide sequences with the lowest MPNN scores were tabulated in Fig. 3D for the next experimental characterization. After sequence optimization with ProteinMPNN, we further refined the peptide candidates using BO, prioritizing sequences with favorable physicochemical properties. Specifically, sequences predicted to have poor aqueous solubility were excluded based on their SWI and FuzzyScore evaluations, ensuring the selection of peptides with both high binding potential and desirable solubility profiles. To gain deeper insights into the binding modes of MBDPs, we analyzed the binding details of the top 3 scoring D-peptides, including MBDP-1, MBDP-2, and MBDP-3. Structural visualizations and interaction analyses revealed strong and specific interactions between these peptides and the MDM2-binding pocket, which collectively highlight the precision and versatility of the design algorithm (Fig. 3E and Fig. S1). In the MBDP-1–target complex, the C-terminal carboxyl group of the peptide forms hydrogen bonds with residues K51 and Y100 of the target protein, significantly enhancing binding stability. Additionally, a hydrogen bond between residue A9 of MBDP-1 and H96 (MDM2) further stabilizes the peptide conformation. Moreover, key hydrophobic residues on the target protein, F55 and M62, form cooperative hydrophobic interactions with the peptide, collectively reinforcing the stability of the protein–peptide interface. In the MBDP-2/MDM2 complex, residues D1 and E2 establish a hydrogen bond network with E69 of MDM2, forming the initial anchoring interface (Fig. S1A). The main chains of E9, I10, and K12 further contribute to binding stability by forming hydrogen bonds with K51, while K12 also interacts with Y100 through an additional hydrogen bond. Moreover, residues A7 and I10 are deeply embedded within the hydrophobic pocket of MDM2, serving as key hydrophobic anchors that reinforce the overall structural complementarity and enhance binding affinity. In the MBDP-3/MDM2 complex, residue D1 maintains a key hydrogen bond interaction with E69 of MDM2, preserving anchoring at the N terminus. At the C terminus, residue S11 forms multiple hydrogen bonds with Y100 and R97, contributing significantly to the binding interface (Fig. S1B). Additionally, S11, E8, A9, and K51 together form a stable hydrogen bond network that further reinforces the interaction. Residue A7 is centrally positioned within the binding pocket and serves as a hydrophobic anchor, enhancing the overall stability of the peptide–protein complex through nonpolar interactions. These diverse yet complementary binding interactions underscore the adaptability and precision of the algorithm in generating mirror peptides optimized for specific receptor interfaces.

To verify that the binding mode of our designed D-peptide to the target protein was consistent with structural predictions, we performed a series of NMR experiments. First, we expressed uniformly ^15^N-labeled, nonliganded MDM2 N-terminal domain (apo-MDM2N) and acquired its ^1^H–^15^N HSQC (heteronuclear single quantum coherence) spectrum. MBDps was synthesized using solid-phase peptide synthesis (SPPS) (Fig. S2). The spectrum was mapped against published data for apo-MDM2 (Fig. S3A) to confirm that the recombinant protein adopted the correct fold [25]. Next, we titrated synthetic MBDP-1 into the ^15^N-labeled apo-MDM2N sample in a concentration-dependent manner until saturation was achieved, and monitored chemical shift perturbations (CSPs) during the process. According to the maximum CSP values (Δδ_max) as shown in Fig. S3B, MBDP-1 was validated to be located in p53-binding site of MDM2 [26]. Furthermore, several MDM2 residues—Y100, F55, K51, and M62—were identified to exhibit significant perturbations in HSQC spectrum in peptide-concentration-dependent manner (Fig. 3F). These results indicate that these residues are directly involved in peptide binding with MDM2. The above experimental results align robustly with our computational predictions, validating Mirror-Peptidizer’s capacity to generate functional D-peptide therapeutics.

### Binding and functional characterization of MBDPs

**Fig. 4.**
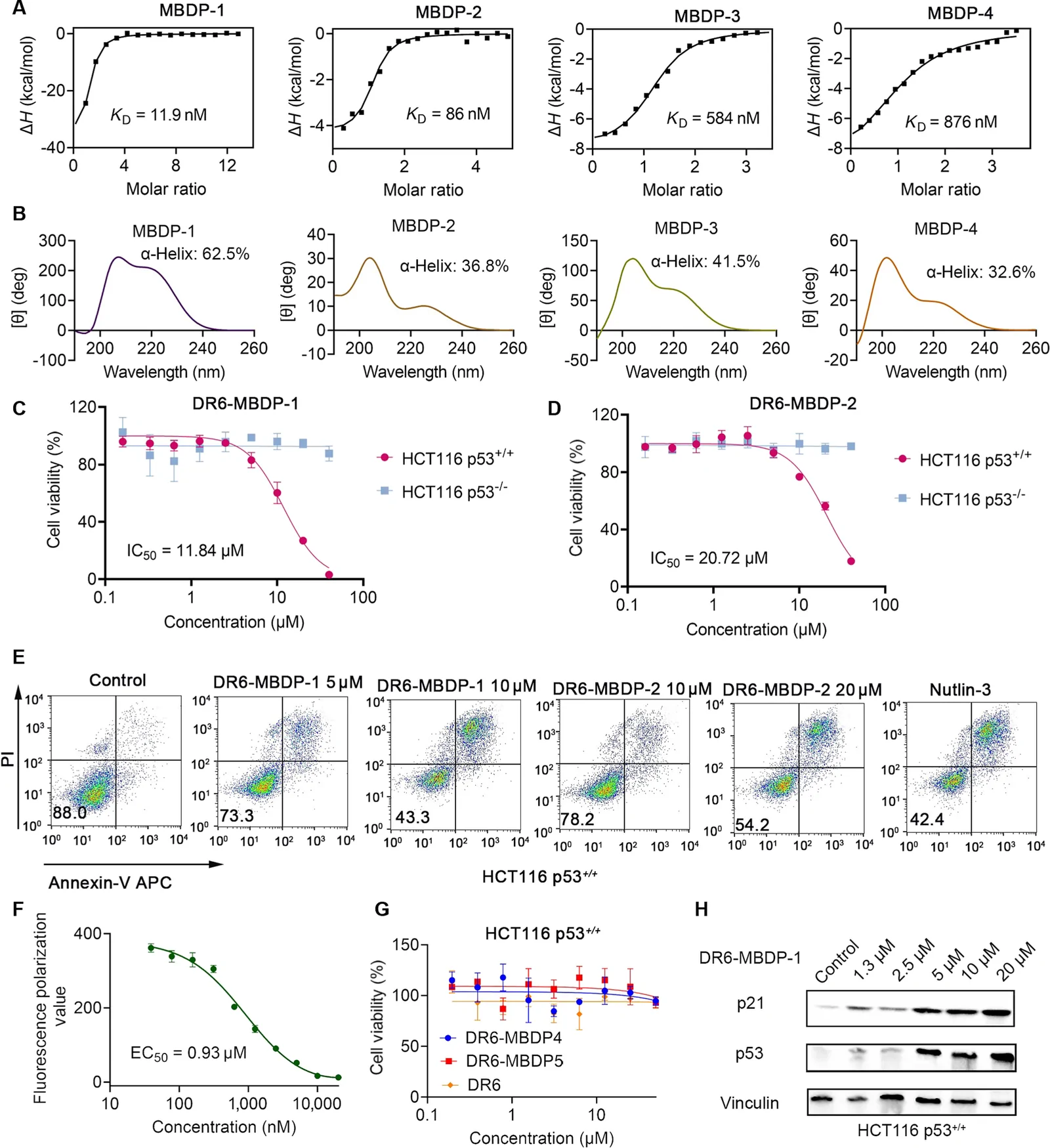
Affinity and biological validation of MDM2-targeting D-peptides. (A) ITC analysis of the binding affinity of MBDP-1–MBDP-4 to MDM2 protein. Representative ITC thermograms and fitted binding isotherms for the interaction between MDM2 and MBDP peptides are shown. Data were fitted using a one-site binding model. The *K*_D_ values indicated in the panels correspond to the fitted values from the representative ITC experiment shown. (B) CD spectroscopy analysis of the secondary structure of MBDP-1–MBDP-4 peptides. MBDP-1, MBDP-2, MBDP-3, and MBDP-4 showed estimated α-helical contents of 62.5%, 36.8%, 41.5%, and 32.6%, respectively. (C and D) Effect of DR6-MBDP-1 and DR6-MBDP-2 on the proliferation of HCT116 p53*^+/+^* and p53^−/−^ cells. (E) Flow cytometry analysis of DR6-MBDP-1- and DR6-MBDP-2-induced apoptosis in HCT116 p53*^+/+^* cells. (F) Fluorescence polarization competition assay showing that MBDP-1 disrupts the interaction between MDM2 and a p53-derived peptide in a concentration-dependent manner. (G) Cell viability analysis of HCT116 p53*^+/+^* cells treated with DR6 alone or control D-peptide conjugates DR6-MBDP4 and DR6-MBDP5. (H) Western blot analysis of p53-pathway activation in HCT116 p53^+/+^ cells treated with increasing concentrations of DR6-MBDP-1. DR6-MBDP-1 induced dose-dependent accumulation of p53 and up-regulation of the canonical p53 target p21. Vinculin was used as the loading control.

To experimentally validate the binding affinity of the generated MBDPs from Mirror-Peptidizer, we synthesized the top 9 mirror-peptide candidates using SPPS. These peptides were then subjected to ITC experiments to assess their binding affinities to the MDM2 protein. The ITC results revealed that 4 of the 9 MBDPs exhibited high binding affinities, with the strongest peptide MBDP-1 achieving an impressive dissociation constant (*K*_D_) of 11.9 nM (Fig. 4A and Fig. S4). The *K*_D_ values of MBDP-2 to MBDP-4 were 86, 584, and 876 nM (Fig. S4), respectively, while remaining MBDP peptides show no affinity to MDM2 (Fig. S5). To further validate the NMR analysis results, we performed site-directed mutagenesis of the identified residues and measured the binding affinities of MBDP-1 with the resulting MDM2 variants using ITC assays (Fig. S6). The results strongly support the binding interface suggested by NMR: The Y100A mutation reduced binding affinity of MBDP-1 to 632 nM, representing a 53-fold decrease relative to the wild-type. The F55A mutation yielded an affinity of 233 nM, a 19-fold decrease. The K51A mutant exhibited a binding affinity of 30.3 nM, corresponding to a 2.5-fold decrease. Of note, a remarkable logarithmic correlation (*R*^2^ = 0.9562) could be observed for the binding potency of MBDPs with the MPNN scores (Fig. S7). To further validate the interaction between MBDP-1 and MDM2 using an orthogonal biophysical method, we performed biolayer interferometry (BLI) with MDM2 and MBDP-1. As shown in Fig. S8, MBDP-1 exhibited clear concentration-dependent binding with well-resolved association and dissociation kinetics. Steady-state analysis yielded a *K*_D_ of 29 nM, which is in good agreement with the ITC-derived affinity. These independent measurements further confirm the high-affinity interaction between MBDP-1 and MDM2 and support the proposed noncanonical binding mode. Collectively, ITC, fluorescence polarization, BLI, NMR CSP, and mutagenesis consistently demonstrate that MBDP-1 specifically recognizes the canonical p53-binding pocket of MDM2 despite adopting a distinct sequence composition from previously reported MDM2-binding peptides.

Circular dichroism (CD) analysis of MBDP-1–MBDP-4 peptides revealed that the D-peptide targeting MDM2 adopted an α-helical structure, which was consistent with the design predictions (Fig. 4B). Quantitative estimation from the CD spectra indicated α-helical contents of 62.5%, 36.8%, 41.5%, and 32.6% for MBDP-1, MBDP-2, MBDP-3, and MBDP-4, respectively. Above findings confirmed the ability of our algorithm to generate high-affinity peptides for MDM2.

Despite the intrinsic proteolytic stability of D-peptides, their high molecular weight often limits their ability to cross cellular membranes, which poses a significant challenge for intracellular targets such as MDM2. To address this issue, we appended a cell-penetrating sequence comprising 6 arginine residues (R6) to the N terminus of the most promising MBDPs. The resulting constructs demonstrated the ability to effectively penetrate cancer cell membranes and target intracellular MDM2. This disruption of the MDM2/p53 interaction restored p53 activity, leading to the induction of apoptosis in cancer cells. We synthesized DR6-MBDP-1 and DR6-MBDP-2 and tested their effects on HCT116 colorectal cancer cells. Treatment with these peptides significantly inhibited the proliferation of HCT116 *p53*^+/+^ cells, confirming their ability to restore p53 activity (Fig. 4C and D). The IC_50_ (median inhibitory concentration) values of DR6-MBDP-1 and DR6-MBDP-2 were 11.84 and 20.72 μM, respectively. Importantly, the peptides exhibited no inhibitory effect on HCT116 *p53*^−/−^ cells, thereby demonstrating the specificity of their mechanism of action and further validating the effectiveness of our drug design approach. We further treated cancer cells with both high and low concentrations of DR6-MBDP-1 and DR6-MBDP-2 to assess their ability to induce apoptosis. Nutlin-3 was used as a positive control (Fig. 4E). To further examine whether the cellular activity of DR6-MBDP-1 was associated with MDM2/p53 pathway engagement, we performed additional biochemical and cellular control experiments. In a fluorescence polarization competition assay, MBDP-1 inhibited the interaction between MDM2 and a p53-derived peptide in a concentration-dependent manner, with a EC_50_ (median effective concentration) value of 0.93 μM, indicating that the MBDP-1 can compete with the p53-binding interface of MDM2 in vitro (Fig. 4F). We next tested DR6 alone and 2 inactive or weakly active control conjugates, DR6-MBDP4 and DR6-MBDP5, in HCT116 p53*^+/+^* cells. These controls showed little effect on cell viability under the tested conditions, suggesting that the observed activity of DR6-MBDP-1 was not due to the DR6 sequence alone or nonspecific toxicity of D-peptide conjugates (Fig. 4G). Finally, Western blot analysis showed that DR6-MBDP-1 induced dose-dependent accumulation of p53 and up-regulation of p21 in HCT116 p53*^+/+^* cells (Fig. 4H). Together, these results support that DR6-MBDP-1 engages the MDM2/p53 pathway and activates p53-dependent signaling in cells.

These results highlight the potential of DR6-MBDPs as a novel therapeutic strategy for cancers with intact p53 function. Moreover, the success of this approach underscores the versatility and reliability of our D-peptide design algorithm, paving the way for its application to other intracellular targets.

### The design of PD-L1-binding D-peptides through Mirror-Peptidizer

**Fig. 5.**
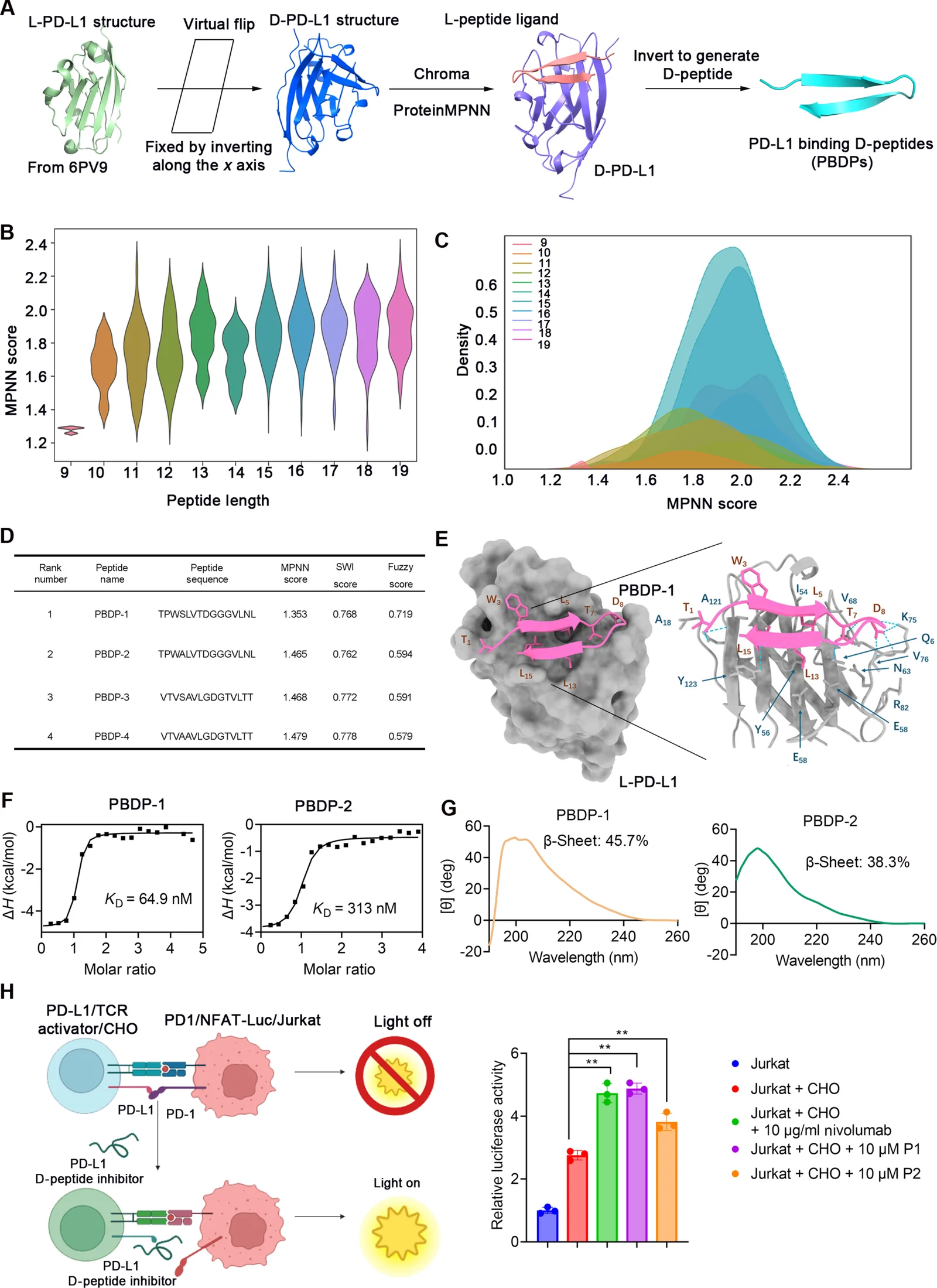
Computational design and validation of D-peptides targeting PD-L1. (A) Schematic overview of the Mirror-Peptidizer pipeline for designing PD-L1-binding D-peptides (PBDPs). (B) Plot of PBDP length versus MPNN design score. (C) Density plots illustrating the distribution of PBDP lengths and MPNN scores. (D) Top 4 PBDP sequences and their corresponding MPNN scores. (E) Detailed structural analysis of PBDP-1 binding to PD-L1 protein. (F) ITC analysis of the binding affinity of PBDP-1–PBDP-2 to PD-L1 protein. Representative ITC thermograms and fitted binding isotherms for the interaction between PD-L1 and PBDP peptides are shown. Data were fitted using a one-site binding model. The *K*_D_ values indicated in the panels correspond to the fitted values from the representative ITC experiment shown. (G) CD spectroscopy analysis of the secondary structure of PBDP-1–PBDP-2 peptides. PBDP-1 and PBDP-2 displayed β-sheet rich profiles, with estimated β-sheet contents of 45.7% and 38.3%, respectively. (H) Detection of PBDP-1–PBDP-2 efficacy in a PD-1/PD-L1 cell luminescent model.

Encouraged by the successful design of MDM2-binding D-peptides, we translated our Mirror-Peptidizer into other important extracellular targets. To this end, we extended our approach to design D-peptides targeting PD-L1, a critical immune checkpoint protein involved in cancer immunotherapy. Similar as above workflow of the design of MBDPs, we used the L-protein structure of PD-L1 (PDB: 6PV9) [27] and applied our algorithm to generate the PD-L1-binding D-peptides, termed PBDPs (Fig. 5A).

The design of PBDPs exhibited uniformity in MPNN scores across sequences of varying lengths. Notably, peptides with a length of 9 amino acids demonstrated a distinct advantage in terms of MPNN evaluation scores (Fig. 5B). This observation suggests that optimal peptide length may vary depending on the target protein, further highlighting the adaptability of our algorithm. Figure 5C illustrated a concentration of lower scores for shorter peptides, particularly at 9 amino acids, indicating an inherent preference for compact sequences in this target system. Despite the overall MPNN scores for PD-L1 designs being worse than those for MDM2, the algorithm successfully produced high-affinity candidates. We presented the top 4 D-peptide sequences with the best scores in Fig. 5D. To understand the binding characteristics of the highest-scoring PBDP-1, we analyzed its complex structure with PD-L1 in detail. The designed D-peptide adopted a β-sheet conformation and formed strong binding interactions with PD-L1 through an extensive hydrogen-bonding network (Fig. 5E). The residue T1 of PBDP-1 formed a hydrogen bond with the receptor residue A121, while L15 established a hydrogen bond with Y123 on PD-L1. Additionally, D8 contributed to the interaction through a hydrogen bond with K75. Beyond these hydrogen bonds, hydrophobic interactions played a significant role in reinforcing the binding. L13 engaged in hydrophobic contacts with Y56, and L5 formed hydrophobic interactions with I54. β-Sheet of PBDP-1 contrasts with the helical structure observed in the MDM2-binding D-peptides, demonstrating our algorithm’s ability to generate diverse structural motifs tailored to different targets. Such flexibility underscores the broad applicability of the method to various protein–protein interaction interfaces.

After solid-phase synthesis of the top 4 PBDP-1 to PBDP-4 (Fig. S9), we tested their binding affinities to PD-L1 using ITC. The results revealed that the top 2 peptides displayed high affinity, with the strongest binding peptide (PBDP-1) achieving a *K*_D_ of 64.9 nM (Fig. 5F and Fig. S10). *K*_D_ value of PBDP-2 was determined to be 313 nM (Fig. 5F and Fig. S10), while PBDP-3 and PBDP-4 showed no affinity to PD-L1 (Fig. S11). In addition, to validate the binding mode of the designed D-peptide targeting PD-L1, we performed site-directed mutagenesis of a key binding residue on PD-L1. Mutation of K75 to alanine (K75A) resulted in a marked reduction in binding affinity, with the dissociation constant increasing from 64.9 nM to 1.03 μM (Fig. S12). This substantial decrease in affinity further confirms the accuracy of our pipeline in predicting the structural interface of the PD-L1/D-peptide complex. These findings further confirm the reliability of our algorithm in generating potent D-peptide candidates for challenging extracellular targets such as PD-L1. Secondary structure analysis further confirmed that PBDP-1 and PBDP-2 adopt a β-sheet conformation, with estimated β-sheet contents of 45.7% for PBDP-1 and 38.3% for PBDP-2, consistent with their predicted β-structured binding modes (Fig. 5G). To evaluate the functional efficacy of PBDP-1 and PBDP-2, we constructed an in vitro cell-based model to assess their ability to inhibit the PD-1/PD-L1 interaction. Remarkably, both peptides effectively blocked PD-1/PD-L1 binding, nivolumab was used as a positive control (Fig. 5H). These results demonstrate the therapeutic potential of the designed D-peptides and highlight their promise as alternatives to existing monoclonal antibody therapies.

In summary, the successful design and validation of PD-L1-binding D-peptides provide strong evidence for the robustness and versatility of Mirror-Peptidizer. The ability to generate peptides with distinct structural motifs and high affinity for diverse targets underscores the potential of this method to revolutionize D-peptide design across a wide range of therapeutic applications.

### The design of IL-23R-binding D-peptides through Mirror-Peptidizer

**Fig. 6.**
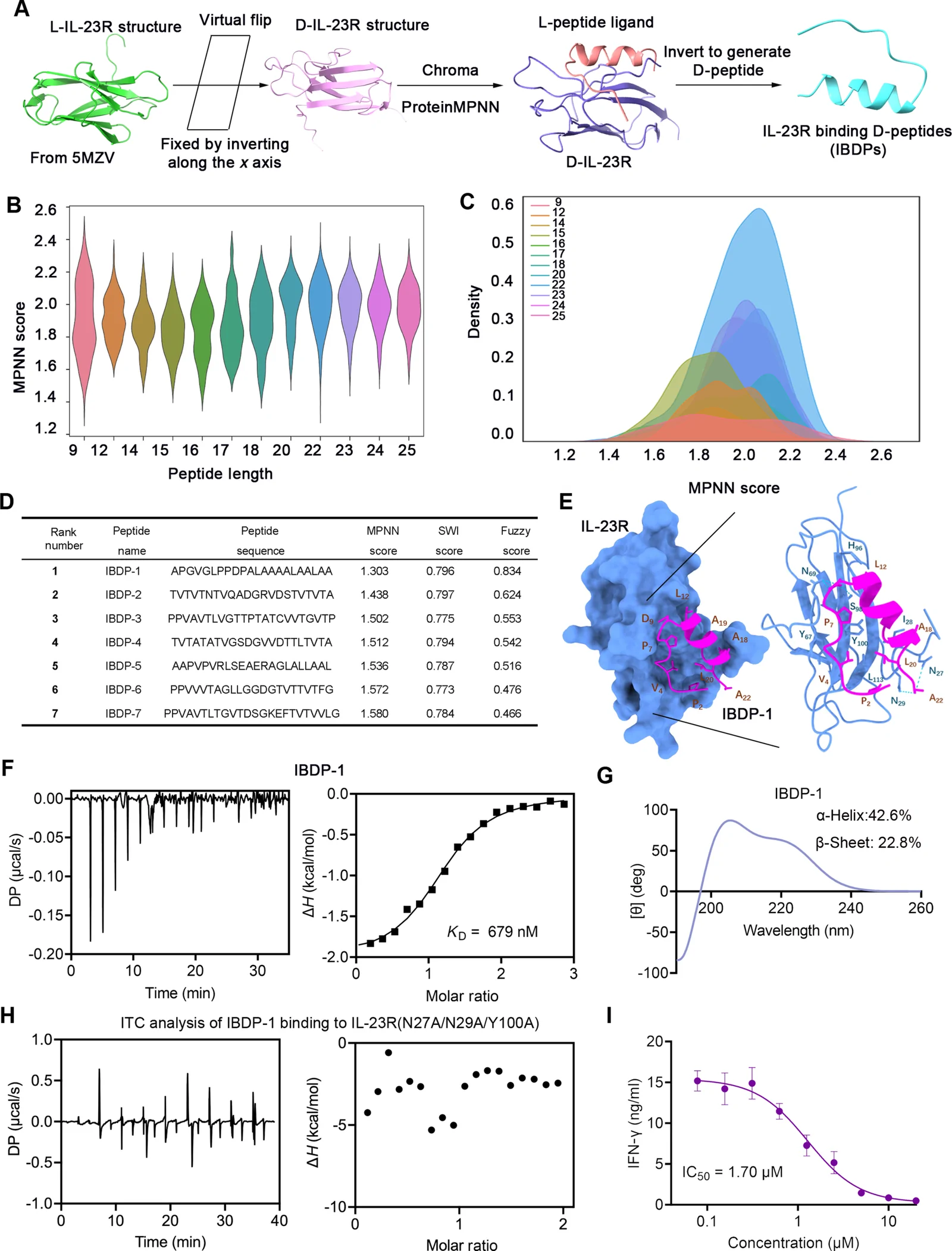
Computational design and validation of D-peptides targeting IL-23R. (A) Schematic overview of the Mirror-Peptidizer pipeline for designing IL-23R-binding D-peptides (IBDPs). (B) Plot of IBDP length versus MPNN design score. (C) Density plots illustrating the distribution of IBDP lengths and MPNN scores. (D) Top 7 IBDP sequences and their corresponding MPNN scores. (E) Detailed structural analysis of IBDP-1 binding to IL-23R protein. (F) ITC analysis of the binding affinity of IBDP-1 to IL-23R protein. Representative ITC thermograms and fitted binding isotherms for the interaction between IL-23R and IBDP-1 are shown. Data were fitted using a one-site binding model. The *K*_D_ value indicated in the panel corresponds to the fitted value from the representative ITC experiment shown. (G) CD spectroscopy analysis of the secondary structure of IBDP-1 peptide. IBDP-1 showed a mixed secondary-structure profile, with 42.6% α-helical content and 22.8% β-sheet content. (H) ITC analysis of IBDP-1 binding to the IL-23R triple mutant IL-23R(N27A/N29A/Y100A). Representative ITC analysis of IBDP-1 binding to IL-23R(N27A/N29A/Y100A) is shown. The triple mutation markedly reduced or abolished detectable binding under the same assay conditions. Because no reliable binding isotherm was observed, *K*_D_ was not determined. (I) Functional evaluation of IBDP-1 in cytokine-stimulated human PBMCs. PBMCs were stimulated with IL-2, IL-12, and IL-23, and IFN-γ production was measured after treatment with increasing concentrations of IBDP-1. Data are shown as mean ± SD, *n* = 3.

To further validate the versatility of our mirror-peptide design algorithm, we selected IL-23R as another target. Using the L-protein structure of IL-23R from Protein Data Bank (PDB) file (PDB: 5MZV) [28], we applied our algorithm to design IL-23R-binding D-peptides, termed IBDPs (Fig. 6A). As observed with MDM2 and PD-L1, the MPNN evaluation scores for different sequences demonstrated good uniformity, indicating consistent optimization across peptide candidates (Fig. 6B). The distribution of MPNN scores across different binder lengths further emphasizes the consistent quality of the designed sequences, with color gradients indicating peptide length (Fig. 6C). We presented the top 7 IL-23R-binding D-peptide sequences in Fig. 6D, highlighting their potential for high-affinity interactions. Among the designed peptides, IBDP-1 emerged as a particularly promising candidate. Detailed structural analysis revealed that IBDP-1 adopts a unique conformation, comprising a partial α-helix coupled with a flexible region (Fig. 6E). The residues of IBDP-1, shown in orange, established key interactions with the receptor residues. Specifically, residue A22 formed hydrogen bonds with receptor residues N27 and N29. D9 formed hydrogen bonds with N69 and S98, while P7 interacted closely with Y100, and P2 engaged with L113. Additionally, IBDP-1 adopts a partial α-helix conformation, with L12 forming hydrophobic interactions with receptor residue H96. This structural profile is distinct from the β-sheet structure observed in PD-L1-binding peptides and the predominantly helical conformation of MDM2-binding peptides, further underscoring the adaptability of our algorithm in generating diverse structural motifs tailored to specific targets.

To experimentally confirm the binding affinity of IBDP-1 to IL-23R, we synthesized the peptide IBDP-1 (Fig. S13) and evaluated its interaction with IL-23R via ITC. The results indicated that IBDP-1 binds to IL-23R with a *K*_D_ value of 679 nM (Fig. 6F). To independently verify the interaction between IBDP-1 and IL-23R, we further performed BLI analysis. As shown in Fig. S14, IBDP-1 bound recombinant human IL-23R in a concentration-dependent manner with clear association and dissociation kinetics. Steady-state fitting yielded a *K*_D_ of 490 nM, which closely agrees with the affinity measured by ITC (*K*_D_ = 679 nM). These orthogonal biophysical data further confirm the direct interaction between IBDP-1 and IL-23R. While this binding affinity is comparatively lower than that observed for MDM2 and PD-L1 targets, it demonstrates the algorithm’s capability to design peptides for challenging receptor-ligand interfaces. Secondary structure analysis further confirmed that IBDP-1 showed a mixed secondary-structure profile, with 42.6% α-helical content and 22.8% β-sheet content, supporting a partially helical and partially flexible conformation (Fig. 6G). To further validate the predicted IL-23R/IBDP-1 interface, we generated an IL-23R triple mutant, IL-23R(N27A/N29A/Y100A), in which 3 predicted contact residues were substituted with alanine. ITC analysis showed markedly reduced or undetectable binding between IBDP-1 and the IL-23R triple mutant, supporting the contribution of these residues to the designed binding interface (Fig. 6H). We next evaluated the functional activity of IBDP-1 in human peripheral blood mononuclear cells (PBMCs). PBMCs were stimulated with IL-2, IL-12, and IL-23 to induce interferon-γ (IFN-γ) production, followed by treatment with increasing concentrations of IBDP-1. IBDP-1 inhibited cytokine-induced IFN-γ production in a concentration-dependent manner, with an IC_50_ of 1.70 μM (Fig. 6I). These results provide additional interface-level and functional evidence for the IL-23R-targeting D-peptide.

The successful design and validation of IL-23R-binding D-peptides, particularly IBDP-1, reinforce the robustness and flexibility of our algorithm. The structural diversity exhibited by the designed peptides across different targets highlights the potential of this approach to address a wide range of therapeutic needs, including those involving complex or less-characterized protein–protein interactions.

### Benchmarking and stereochemical quality control of mirror-derived design

Overall, the Mirror-Peptidizer workflow integrates de novo backbone generation, sequence design, and local sequence optimization into a unified computational pipeline for D-peptide discovery. Across the 3 representative targets, Chroma generated a structurally diverse ensemble of peptide backbones across the 3 representative targets rather than converging on a single predefined binding mode. After surface-geometry quality control, 225, 194, and 158 backbones were retained for MDM2, PD-L1, and IL-23R, respectively, and these candidates displayed distinct peptide-length distributions, secondary-structure compositions, and receptor-contact patterns. Representative structures, including the experimentally prioritized candidates and additional scaffolds from distinct structural clusters, further showed that the designed peptides occupied different binding poses and adopted target-dependent backbone conformations. The complete design funnel, structural-diversity analysis, and backbone-level descriptors are provided in Table S1, Fig. S15, and Data S1. ProteinMPNN subsequently generated approximately 4,000 candidate sequences per target, which were further filtered based on structural compatibility and sequence complexity to remove redundant or unfavorable designs (Table S2). BO then performed local refinement of the retained candidates through limited sequence substitutions, simultaneously improving backbone compatibility and predicted synthetic accessibility while largely preserving the original sequence framework (Table S3). This hierarchical design strategy reduced the search space from thousands of computational candidates to a small number of experimentally prioritized peptide series (MBDP, PBDP, and IBDP), providing an efficient and reproducible framework for artificial intelligence (AI)-guided de novo D-peptide discovery.

To examine whether mirror-derived D-peptide design explores a sequence and structural space distinct from direct L-peptide design, we performed matched D/L design analyses on PD-L1, MDM2, and IL-23R. For each target, direct L-peptide design was performed on the native L-target, whereas mirror-derived D-peptide design was performed by generating L-peptide binders against the mirrored D-target followed by coordinate mirroring back to the native L-target frame. Structurally abnormal Chroma poses were removed before analysis, and L-peptide candidates were down-sampled to match the corresponding D-peptide-derived candidate numbers.

The 2 design modes produced clearly different candidate distributions. The median ProteinMPNN scores of mirror-derived D-peptide candidates were 1.912, 1.951, and 1.852 for PD-L1, MDM2, and IL-23R, respectively, whereas the corresponding values for direct L-peptide candidates were 1.622, 1.704, and 1.606. Kolmogorov–Smirnov tests confirmed significant distributional differences for all 3 targets (PD-L1: KS = 0.472, *P* < 0.0001; MDM2: KS = 0.452, *P* < 0.0001; IL-23R: KS = 0.545, *P* < 0.0001) (Fig. S16A). Consistently, t-SNE analysis of the top 100 candidates showed separation between mirror-derived D-peptide and direct L-peptide sequence spaces (Fig. S16B). We next compared representative structures by transforming the mirror-derived complexes back into the native L-target coordinate frame. The mirror-derived D-peptide candidates differed from direct L-peptide candidates in binding pose, backbone trajectory, and secondary-structure arrangement (Fig. S16C), indicating that the mirrored target imposes distinct geometric constraints during Chroma backbone generation.

To further validate stereochemical consistency after mirroring, we performed a post-generation backbone-chirality quality-control analysis based on paired L- and D-backbone dihedral angles. All paired structures showed the expected mirror relationship, with maximum |φL + φD| and |ψL + ψD| values of 0.000° across 32,116 paired φ and 32,116 paired ψ angles. The signed N-Cα-C-Cβ scalar triple product was also checked for nonglycine residues to confirm stereochemical inversion and identify records requiring inspection. These analyses verify that the deterministic mirror transformation correctly inverts backbone handedness and preserves dihedral consistency (Fig. S16D) while not implying that Chroma or ProteinMPNN explicitly models chirality.

To evaluate the contribution of individual modules, we compared 3 workflows on PD-L1, MDM2, and IL-23R: Chroma + ProteinMPNN, Chroma + BO, and the complete Chroma + ProteinMPNN + BO workflow. ProteinMPNN score was used as a lower-is-better sequence–backbone compatibility metric, whereas synthesis penalty was used as a lower-is-better estimate of synthesis-related risk.

Chroma + ProteinMPNN achieved the lowest ProteinMPNN scores across all 3 targets, but its top candidates showed high or moderate synthesis penalties. In contrast, Chroma + BO reduced synthesis penalties to the low-risk range, but at the cost of substantially worse ProteinMPNN scores. The complete Chroma + ProteinMPNN + BO workflow provided a more balanced outcome, maintaining acceptable ProteinMPNN scores while reducing synthesis penalties to the low-risk range for all 3 targets (Fig. S17 and Table S4).

These results indicate that ProteinMPNN and BO play complementary roles: ProteinMPNN prioritizes sequence–backbone compatibility, whereas BO improves synthetic tractability. Thus, the integrated workflow serves as a multi-objective candidate-prioritization strategy for selecting structurally plausible and experimentally tractable D-peptide candidates.

To further evaluate the computational feasibility of Mirror-Peptidizer beyond the 3 benchmark targets, we first quantified the number of candidates retained after post-generation QC (quality control) filtering. For the benchmark targets, 1,808 of 4,000 PD-L1 candidates, 2,007 of 4,000 MDM2 candidates, and 1,266 of 3,990 IL-23R candidates passed the QC filters, corresponding to retention rates of 45.2%, 50.2%, and 31.7%, respectively.

We next applied the same Tier-1 workflow and QC criteria to 3 additional targets: tumor necrosis factor-α (TNF-α), CXCR2, and CXCR4. These targets were selected to represent different structural contexts, including a soluble cytokine and chemokine receptors. After QC filtering, 340 of 3,990 TNF-α candidates, 1,757 of 3,990 CXCR2 candidates, and 1,911 of 3,990 CXCR4 candidates were retained, corresponding to retention rates of 8.5%, 44.0%, and 47.9%, respectively.

The retained candidates were further ranked by ProteinMPNN score. Among the additional targets, CXCR2 and CXCR4 showed more favorable score profiles than TNF-α. The best ProteinMPNN scores were 1.4584 for CXCR2 and 1.3946 for CXCR4, compared with 2.0915 for TNF-α. Similarly, the top-100 mean scores were 1.6745 and 1.5681 for CXCR2 and CXCR4, respectively, compared with 2.2764 for TNF-α (Table S5). These results suggest that Mirror-Peptidizer can generate QC-passed and rankable candidates across additional targets, although the efficiency and score distribution are target-dependent. Overall, 9,089 of 23,960 candidates passed the updated computational QC filters, supporting the feasibility of the workflow while highlighting the need for downstream BO optimization, orthogonal interface scoring, and experimental validation.

Although these analyses support the geometric and stereochemical consistency of the mirror-coordinate workflow, they do not indicate that Chroma or ProteinMPNN explicitly models chirality; therefore, Mirror-Peptidizer should be interpreted as a proof-of-concept mirror-coordinate design workflow rather than a fully chirality-aware generative model.

## Discussion

D-peptides, composed of D-type amino acids that do not exist naturally, are the mirror images of L-peptides [29,30]. Building on the inherent advantages of peptide drugs, such as their high affinity for target proteins, D-peptides offer an additional benefit: They are not recognized by L-type proteasomes and are therefore resistant to degradation, resulting in an extended half-life [31]. The notable example was Parsabiv approved by the U.S. Food and Drug Administration (FDA) for treating secondary hyperparathyroidism, which comprised 7 D-amino acids and thus demonstrated greater potency than its L-counterpart [32,33]. It was worth noting that, since the first report of mirror-image phage display nearly 30 years ago, only a few mirror-image peptides have been developed against numerous protein targets [34,35].

D-peptide discovery has traditionally relied on mirror-image phage display, in which chemically synthesized D-protein targets are used to screen genetically encoded L-peptide libraries, followed by enantiomeric conversion of the selected ligands. This strategy elegantly addresses the chirality problem and has yielded numerous D-peptide binders, but it depends on the availability of correctly folded D-protein targets and experimental screening [36,37]. Recent advances in chemical protein synthesis and D-protein-assisted ligand discovery have further expanded access to mirror-image targets and enabled the identification of D-peptide and macrocyclic D-peptide binders against disease-relevant proteins [38]. Nevertheless, these approaches still require chemical preparation of mirror-image proteins and experimental selection.

Recent computational studies have provided alternative routes for heterochiral binder design [39]. Coordinate mirroring strategies have been used to generate D-peptide ligands without requiring physical D-protein targets, whereas de novo design approaches combined with experimental validation have enabled the generation of heterochiral D-protein and D-miniprotein binders against native L-protein targets [13]. These studies provide important conceptual support for exploiting mirror-coordinate transformations in heterochiral molecular recognition. However, many such approaches focus on folded proteins or miniproteins and often rely on large-scale screening or affinity maturation.

In parallel, emerging D-peptide-specific computational methods have highlighted the need for chirality-aware or chirality-adapted modeling. Receptor-conditioned generative models, structure-based representations, and mirror-image training strategies have been explored to address the stereochemical challenges unique to D-peptides. At the same time, recent analyses suggest that general-purpose structure prediction tools such as AlphaFold 3 may not reliably predict D-peptide chirality, fold, or binding poses, indicating that heterochiral peptide modeling remains an unsolved problem.

Within this context, Mirror-Peptidizer should be viewed as a practical mirror-coordinate workflow for generating experimentally testable D-peptide candidates directly from native L-protein structures. Rather than replacing mirror-image phage display or explicitly chirality-aware models, the method removes the need for D-protein synthesis during the initial candidate-generation stage by combining coordinate mirroring, Chroma-based backbone generation, ProteinMPNN-based sequence assignment, and BO. Therefore, Mirror-Peptidizer represents an incremental and pragmatic strategy for D-peptide candidate generation rather than a universal solution to heterochiral peptide design.

To validate the effectiveness of Mirror-Peptidizer, we applied it to 3 therapeutically relevant targets: MDM2, PD-L1, and IL-23R. The algorithm successfully generated D-peptides with diverse structural conformations—helix for MDM2-binding peptides (MBDPs), β-sheet for PD-L1-binding peptides (PBDPs), and a mixed α-helix and flexible structure for IL-23R-binding peptides (IBDPs). These peptides demonstrated binding potencies ranging from low nanomolar to sub-micromolar levels, underscoring the robustness of the algorithm across various protein–protein interaction interfaces. The variability in peptide structures arises from the differing topologies of their target proteins. MDM2 contains a deep hydrophobic groove with a “keyhole”-like architecture that naturally favors ligands capable of adopting a stable α-helical structure [40]. Accordingly, the designed peptide, MBDP-1, adopts an α-helical conformation to achieve optimal shape complementarity with the binding site. In contrast, PD-L1 presents a relatively flat and open binding surface [41]. To accommodate this topology, the algorithm generated a β-sheet-forming peptide, PBDP-1, which spans the target surface and maximizes binding through an extensive hydrogen-bonding network. IL-23R, on the other hand, features a shallow and irregular cleft that necessitates greater conformational flexibility in its ligand [42]. In response to this structural challenge, the designed peptide, IBDP-1, adopts a mixed conformation comprising partial α-helix and random coil elements, enabling it to effectively insert into and conform to the complex binding interface.

Notably, the MDM2-binding D-peptides conjugated with cell-penetrating peptides (R6-MBDPs) exhibited the ability to penetrate cancer cells and effectively inhibit the MDM2/p53 interaction, leading to p53-dependent apoptosis. Similarly, the PD-L1-binding D-peptides (PBDPs) successfully blocked the interaction of PD-1 and PD-L1 in vitro, achieving an efficacy comparable to that of clinically approved immune checkpoint inhibitors. These findings highlight the potential of Mirror-Peptidizer to generate functional D-peptides with therapeutic relevance.

A limitation of the current implementation is that the ranking function does not explicitly calculate the intermolecular binding energy between the final D-peptide and its native L-protein target. The ProteinMPNN-derived score primarily evaluates sequence compatibility with the designed backbone environment, whereas the synthesis-related term evaluates sequence tractability. Consequently, the highest-ranked candidates should be interpreted as prioritized experimentally testable sequences rather than globally optimal binding solutions. Incorporating interface-specific energetic calculations, molecular-dynamics-based stability evaluation, and experimentally trained affinity-prediction models represents an important direction for future development of the Mirror-Peptidizer framework.

In summary, Mirror-Peptidizer represents a significant advancement in D-peptide design, demonstrating its ability to produce high-affinity peptide binders with diverse structural motifs tailored to different protein targets. By addressing key challenges in D-peptide development, this pipeline holds promising potential to revolutionize D-peptide therapeutics across a broad spectrum of applications, including oncology, immunotherapy, and beyond.

## Materials and Methods

### Materials

All Fmoc-protected D-amino acids were purchased from GL Biochem. Rink-amide-MBHA resin (loading 0.55 to 0.75 mmol/g) was purchased from Tianjin Nankai Hecheng Science & Technology Co. Ltd. (Tianjin, China). Trifluoroacetic acid (TFA; Aladdin Chemicals, catalog no. T103295), tris(2-carboxyethyl)phosphine hydrochloride (TCEP; Sigma-Aldrich, catalog no. C4706), reduced glutathione (GSH; Sigma-Aldrich, catalog no. G4251), dichloromethane (DCM; Sigma-Aldrich, catalog no. 270997), N,N-dimethylformamide (DMF; Sigma-Aldrich, catalog no. 227056), high-performance liquid chromatography (HPLC)-grade acetonitrile (Sigma-Aldrich, catalog no. 34851), triisopropylsilane (TIPS; Sigma-Aldrich, catalog no. 8.41359), N,N-diisopropylethylamine (DIPEA; Sigma-Aldrich, catalog no. D125806), thiophenol (Sigma-Aldrich, catalog no. T32808), p-cresol (Sigma-Aldrich, catalog no. C85751), and guanidine hydrochloride (Sigma-Aldrich, catalog no. G4505) were purchased from Sigma-Aldrich. McCoy’s 5A medium (Procell, catalog no. PM150710) and fetal bovine serum (FBS; Procell, catalog no. 164210) were obtained from Procell Life Science & Technology Co. Ltd. (Wuhan, China). The Annexin V Apoptosis Detection Kit (BioLegend, catalog no. 640932) was purchased from BioLegend.

### D-peptide design algorithm

#### Conversion of the receptor from *L* to *D*

Starting with the coordinates of the L-form receptor $\left\{\left(x_{i},y_{i},z_{i}\right)\right\}$, we apply a coordinate reflection along the *x* axis to obtain the D-form receptor:

$$\left(x_{i},y_{i},z_{i}\right)\;\overset{\;L\to D\;}{\to }\;\left(-x_{i},y_{i},z_{i}\right)$$ (1)

Let the resulting coordinates be denoted as $\left(X_{R}^{D}=\right.\left.\left\{\left(-x_{i},y_{i},z_{i}\right)\right\}\right)$

Our implementation follows the convention adopted by Garton et al. [43] for constructing the mirror-image Protein Data Bank [(D)-PDB], in which all structures were reflected along the *x* axis. We note that this convention is not mathematically unique. Reflection about any single Cartesian axis is an improper orthogonal transformation (determinant = −1), and the 3 possible axis reflections generate equivalent mirror-image structures differing only by rigid-body rotation.

#### Generating the binder backbone under fixed D-form receptor coordinates using a diffusion model (Chroma)

In the subsequent diffusion-based sampling process, the coordinates of the D-form receptor remain fixed, i.e.,

$$\frac{\partial X_{R}^{D}}{\partial t}=0$$ (2)

With $X_{R}^{D}$​ serving as an invariant structural reference, we invoke Chroma to sample the backbone coordinates of the binder:

$$X_{B}=\text{Chroma}\left(X_{R}^{D}\right)$$ (3)

Concatenating the receptor and binder coordinates yields the D-form complex:

$$X_{\text{complex}}^{D}=\left[X_{R}^{D};X_{B}\right]$$ (4)

#### Optimizing the binder sequence using ProteinMPNN

With a stable D-form complex structure in hand, we then use ProteinMPNN to optimize the amino acid sequence of the binder:

$$S_{B}=\text{ProteinMPNN}\left(X_{\text{complex}}^{D}\right)$$ (5)

#### Generating the D-form binder

Finally, after obtaining the optimized L-form binder coordinates, we apply the same coordinate reflection to produce the D-form binder:

$$\left(x_{i},y_{i},z_{i}\right)\;\overset{\;L\to D\;}{\to }\;\left(-x_{i},y_{i},z_{i}\right)$$ (6)

### The multi-objective optimization for peptide candidates

The multi-objective optimization is employed to balance binding affinity/structural stability and peptide solubility, thereby avoiding the generation of candidates with poor physicochemical properties. Rather than using a simple weighted-sum approach, we adopt a desirability-based scoring framework, described as follows:

ProteinMPNN score: This score serves as a proxy for both binding affinity and structural stability. Lower values indicate more favorable interactions. A desirability function is applied to map the raw score linearly into the normalized range [0, 1].

SWI: This metric estimates peptide solubility. For a peptide of length *L*, SWI is calculated as follows: $\mathrm{SWI}\left(S\right)=\frac{1}{L}\sum_{i=1}^{L}\omega \left(s_{i}\right)$. $\omega \left(s_{i}\right)$ is the solubility weight of the *i*th amino acid. Higher SWI values indicate better solubility. The raw SWI is also normalized via a desirability function.

To combine these 2 objectives, we use importance-weighted desirability scores calculated by the following: $O_{j}=D_{j}\left(x\right)+\left(1-D_{j}\left(x\right)\right)\left(1-w_{\mathrm{imp},j}\right)$, where $D_{j}\left(x\right)$ is the desirability value, $O_{j}$ is the weighted score, and $w_{\mathrm{imp},j}$ is the importance factor of the objective j (range, 0 to 1).

Finally, the overall FuzzyScore is computed as the geometric mean of the weighted desirability scores: $\text{FuzzyScore}={\left(\prod_{j=1}^{N}O_{j}\right)}^{1/N}$. In our implementation, *N* = 2 (ProteinMPNN and SWI).

### Detailed implementation parameters of Mirror-Peptidizer

For the benchmark targets PD-L1, MDM2, and IL-23R, candidate generation was performed under predefined target- and peptide-length settings. The same length-specific settings were used for mirror-derived D-peptide design and direct L-peptide design to avoid confounding by peptide length. Approximately 4,000 Chroma-derived candidates were generated for each target and design mode before post-generation quality control. Poses with abnormal backbone geometry, distorted secondary-structure organization, buried placement, or implausible target interfaces were excluded before ProteinMPNN scoring, BO, and D/L comparative analysis. The quality-control step excluded 33, 12, and 40 abnormal Chroma poses for PD-L1, MDM2, and IL-23R, respectively. After this filtering step, the Chroma + ProteinMPNN workflow retained 3,670, 3,880, and 3,590 candidates for PD-L1, MDM2, and IL-23R, respectively.

For the module-level ablation analysis, 3 workflows were evaluated under the same target-specific settings: Chroma + ProteinMPNN without BO, Chroma + BO without ProteinMPNN initialization, and the complete Chroma + ProteinMPNN + BO workflow. The retained candidate numbers and best score/penalty values for each workflow are summarized in Table S1. In the BO workflows, optimization was initialized from the retained Chroma-derived candidate pool, new candidates were proposed in fixed-size rounds, and optimization was terminated after the predefined number of rounds rather than by manual inspection. The BO fitness was computed from the lower-is-better ProteinMPNN score and a synthesis-penalty term, with synthesis-aware filtering and ranking used to avoid candidates predicted to be difficult to synthesize. FuzzyScore was used as a desirability-based prioritization utility: ProteinMPNN score and SWI/synthesis-related terms were mapped to normalized desirability values using fixed bounds and importance factors, and the same settings were applied consistently within each analysis so that candidate ranking reflected sequence-backbone compatibility and predicted synthetic tractability rather than direct binding-affinity prediction.

### Geometry-based pose filtering and post-generation quality control

After Chroma backbone generation, generated binder poses were subjected to geometry-based quality control before downstream sequence assignment and scoring. First, mirror-coordinate conversion was performed by applying a deterministic coordinate inversion to the target or complex coordinates. To remove geometrically implausible Chroma outputs, an optional surface-pose filter was applied to reject binder poses that were buried inside the receptor, poorly positioned relative to the target surface, or lacked sufficient target contact. This filter evaluated radial placement relative to the receptor, local receptor enclosure around the binder, the distribution of occupied octants surrounding the binder, and the number of binder atoms within the predefined contact cutoff. Poses failing these criteria were excluded from subsequent analysis.

After sequence substitution, PDB repair and energy minimization were performed to correct local geometry and remove obvious structural artifacts. Generated peptide backbones were also inspected for abnormal geometry, distorted secondary structures, or poorly formed target interfaces. Structures with clearly abnormal backbone geometry or implausible binding interfaces were removed before downstream ProteinMPNN scoring, BO, and D/L comparative analysis. In the benchmark datasets, this quality-control procedure excluded 33, 12, and 40 abnormal Chroma poses for PD-L1, MDM2, and IL-23R, respectively. These filtered poses were treated as failure cases of the current workflow and were not included in subsequent candidate ranking.

### Peptide synthesis

Peptides were synthesized using SPPS on Rink amide MBHA resins, employing the Fmoc (9-fluorenylmethyloxycarbonyl) chemistry. The synthesis was performed using an optimized HBTU [O-(benzotriazol-1-yl)-N,N,Nʹ,Nʹ-tetramethyluronium hexafluorophosphate] activation and DIPEA in situ neutralization protocol. In this method, Fmoc-protected amino acids were sequentially coupled to the resin-bound peptide chain in the presence of HBTU as a coupling reagent, with DIEA (diisopropylethylamine) as a base to neutralize the released proton during activation. The peptide elongation was performed stepwise, with each amino acid being incorporated after deprotection of the Fmoc group using 20% piperidine in DMF. Each coupling cycle involved a 2-h reaction time to ensure efficient peptide bond formation. After the completion of the peptide elongation, the peptide was cleaved from the resin using a strong cleavage cocktail consisting of TFA, TIPS, and water in a 95:2.5:2.5 ratio. This cleavage process removes the peptide from the solid support while simultaneously deprotecting the side-chain groups of the amino acids, except for any protective groups that require additional treatments. Following cleavage, the crude peptide was precipitated by adding cold diethyl ether to the TFA solution, which facilitated the removal of the solvent and other nonpeptide impurities. The crude peptide was then purified using preparative C18 reversed-phase HPLC. The purification was performed under conditions optimized for the peptide’s hydrophobicity, ensuring the separation of the target peptide from any residual impurities or incomplete sequences. Fractions containing the pure peptide were collected, and solvent was removed by lyophilization. Finally, the molecular masses of the purified peptides were determined using electrospray ionization mass spectrometry (ESI-MS) to confirm the identity and purity of the final products.

### CD spectroscopy

The CD spectra of the peptides were recorded to investigate their secondary structure. CD measurements were performed in phosphate buffer (PB) at a final peptide concentration of 20 μM. To facilitate the formation of secondary structures, 15% trifluoroethanol (TFE) was included in the buffer solution, as TFE is known to promote the stabilization of helical structures in peptides. Spectra were collected using a Jasco J-1500 spectropolarimeter at 25 °C, a temperature that was maintained using a Peltier temperature control unit to ensure consistency during data acquisition. The CD spectra were recorded over a wavelength range of 260 to 190 nm, which covers the characteristic absorbance regions for peptide secondary structures. Measurements were made at 0.2-nm intervals, ensuring high-resolution data acquisition. Each spectrum was the result of an average of 3 scans, and the data were corrected for background absorbance from the buffer solution. The spectral data were processed to determine the molar ellipticity (θ) as a function of wavelength.

### ITC analysis

ITC measurements were performed using a MicroCal PEAQ-ITC instrument (Malvern Panalytical, UK) at 25 °C. Recombinant proteins and peptides were prepared in the same buffer consisting of 20 mM tris–HCl with 150 mM NaCl (pH 7.5). Protein samples were dialyzed extensively against the assay buffer, and peptide solutions were prepared in the identical buffer to minimize heats of dilution. Prior to each experiment, all samples were centrifuged at 3,000*g* for 10 min.

For the MDM2-binding measurements, recombinant human MDM2 was loaded into the sample cell at 5 μM for MBDP-1, 15 μM for MBDP-2, and 20 μM for MBDP-3 to MBDP-7. The corresponding peptides were loaded into the injection syringe at 375 μM for MBDP-1, 435 μM for MBDP-2, 400 μM for MBDP-3, 420 μM for MBDP-4, 440 μM for MBDP-5, 400 μM for MBDP-6, and 460 μM for MBDP-7. For analysis of the predicted MBDP-1-binding interface, MDM2(Y100A), MDM2(F55A), and MDM2(K51A) were loaded into the sample cell at 20 μM, while MBDP-1 was loaded into the syringe at 560, 460, and 230 μM, respectively.

For the PD-L1-binding measurements, recombinant human PD-L1 was loaded into the sample cell at 15 μM for PBDP-1 and 20 μM for PBDP-2, PBDP-3, and PBDP-4. PBDP-1 was loaded into the syringe at 420 μM, whereas PBDP-2, PBDP-3, and PBDP-4 were loaded at 440 μM. Binding of PBDP-1 to the PD-L1(K75A) mutant was examined using 20 μM mutant protein in the sample cell and 580 μM PBDP-1 in the syringe.

For the IL-23R-binding measurements, recombinant human IL-23R was loaded into the sample cell at 20 μM, and IBDP-1 was loaded into the syringe at 320 μM. The IL-23R(N27A/N29A/Y100A) mutant was tested using 20 μM mutant protein in the cell and 230 μM IBDP-1 in the syringe.

Each titration consisted of an initial 0.4-μl injection followed by 19 successive 2.0-μl injections at 120-s intervals, with continuous stirring at 1,000 rpm. The heat generated by the initial injection was excluded from data fitting. Control titrations of each peptide into the corresponding assay buffer were conducted under identical conditions.

The integrated heat signals were analyzed using the MicroCal PEAQ-ITC Analysis Software employing a one-set-of-sites binding model. Binding curves that failed to exhibit a concentration-dependent saturation profile were considered to show no measurable interaction under the experimental conditions and were therefore not fitted for affinity determination.

### BLI analysis

Binding affinities between the designed D-peptides and their target proteins were determined by BLI using an Octet R8 instrument (Sartorius/ForteBio, Germany) equipped with streptavidin (SA) biosensors. Recombinant human MDM2 or IL-23R extracellular domain proteins were biotinylated using an NHS (N-hydroxy succinimide)-biotin reagent according to the manufacturer’s protocol, and excess free biotin was removed by desalting prior to immobilization. SA biosensors were hydrated in running buffer for 10 min before use and equilibrated for 60 s to establish the baseline. Biotinylated proteins were then immobilized onto the biosensors for 180 s, followed by a second baseline prior to kinetic measurements.

Binding experiments were performed in PBST (phosphate-buffered saline supplemented with 0.05% Tween 20) at 30 °C using 96-well microplates. The designed D-peptides (MBDP-1 for MDM2 and IBDP-1 for IL-23R) were serially diluted in a 3-fold concentration series (3,000, 1,000, 333, 111, 37, 12.3, and 4.1 nM). Association was monitored for 180 s, followed by dissociation in running buffer for 120 s. Buffer-only reference wells were included for background subtraction, and each concentration was analyzed using freshly loaded biosensors.

Sensorgrams were processed by reference subtraction and analyzed using Octet Data Analysis v11.1.1.8 (Sartorius). Kinetic parameters were obtained by global fitting to a 1:1 Langmuir binding model.

### Cell viability assay by CCK-8

Cell viability was assessed using the cell counting kit-8 (CCK-8) assay, a colorimetric method that quantitatively measures the metabolic activity of cells as an indicator of cell viability and proliferation. The assay is based on the reduction of the CCK-8 reagent by viable cells, generating a colorimetric product that can be detected spectrophotometrically. HCT116 cells were seeded in 96-well plates at a density of 3 × 10^3^ cells per well in McCoy’s 5A medium supplemented with 10% FBS to ensure optimal cell growth. For each experimental condition, 3 independent samples were seeded in duplicate to account for biological variability. After seeding, cells were allowed to adhere and grow for 24 h under standard culture conditions. Following this incubation period, cells were treated with varying concentrations of R6-MBDP-1 or R6-MBDP-2 peptides and incubated for an additional 48 h. After the treatment period, the cell viability was measured by adding 10 μl of the CCK-8 reagent to each well. The reagent was incubated with cells for 2 h at 37 °C to allow sufficient reduction of the tetrazolium salt. After the incubation, the absorbance of each well was measured at 450 nm using a microplate reader. The absorbance values are directly proportional to the number of viable cells, as metabolically active cells reduce the CCK-8 reagent, generating a colored formazan product. The viability of the cells was calculated as the ratio of the absorbance of treated cells relative to the absorbance of control cells, which were treated with the same concentration of solvent or buffer but without peptide treatment. Results were expressed as percentage cell viability relative to the untreated control group (100% viability).

### Apoptosis assay by flow cytometry

To assess the apoptosis-inducing effect of R6-MBDP-1 and R6-MBDP-2, HCT116 cells were treated for 48 h with the peptides, with nutlin-3 as a positive control and untreated cells as a negative control. After treatment, cells were collected, stained with allophycocyanin (APC)-labeled anti-annexin V and propidium iodide (PI) from the apoptosis detection kit (BioLegend), and analyzed by flow cytometry. The percentage of apoptotic cells (early and late apoptosis) was determined by assessing annexin V and PI fluorescence, with early apoptotic cells identified as annexin V-positive/PI-negative and late apoptotic cells as annexin V-positive/PI-positive. Flow cytometry results were compared to control groups to evaluate the pro-apoptotic effects of the peptides.

### Protein expression and purification for NMR

^15^N-labeled MDM2 (residues 1 to 118) was expressed in *Escherichia coli* Rosetta (DE3) pLysS cells grown in phosphate-based minimal medium supplemented with ^15^NH_4_-Cl as the sole nitrogen source. After cell harvest, the pellet was resuspended in lysis buffer consisting of 25 mM tris–HCl (pH 7.5), 5 mM dithiothreitol (DTT), 1 mM phenylmethylsulfonyl fluoride (PMSF), 1 mM ethylenediaminetetraacetic acid (EDTA), 1 mM benzamidine, 20 mM NaCl, and protease inhibitor cocktail (Sigma-Aldrich). The suspension was sonicated on ice, and the lysate was clarified by centrifugation at 40,000*g* for 30 min at 4 °C. The supernatant was filtered through a 0.45-μm membrane (Millipore) and loaded onto a 20-ml Q-Sepharose anion exchange column (Pharmacia) at a flow rate of 1 ml/min.

The protein was purified using Ni-NTA affinity chromatography, followed by size exclusion chromatography, to obtain the final pure product. The purified MDM2 was used for subsequent NMR studies.

### NMR spectroscopy

All NMR experiments were carried out at 288 K on a Bruker Avance HD-III 600 MHz NMR spectrometer equipped with a 5-mm CPTCI probe. ^1^H-^15^N HSQC spectra were acquired with a Bruker standard HSQC-ETFPF3GPSI pulse sequence and repetition delay of 1.0 s. The direct and indirect acquisition times were 106 and 78 ms, respectively. Data were processed and analyzed using NMRPipe and NMRFAM-SPARKY software.

CSPs were calculated from the differences in ^1^H and ^15^N chemical shifts between the free and ligand-bound states of the protein using the following weighted formula:

$$\Delta \delta =\sqrt{\frac{1}{2}\left[{\left(\delta_{H}-\delta_{H0}\right)}^{2}+0.14{\left(\delta_{N}-\delta_{N0}\right)}^{2}\right]}$$ (7)

where δH0 and δN0 are the proton and nitrogen chemical shifts of the free protein, respectively. The data fitting was done by the Levenberg−Marquardt least-squares algorithm as implemented in the LsqFit package of the Julia programming language.

### PD-1/PD-L1 cell-based luminescence reporter assay

The inhibitory activity of PD-L1-targeting D-peptides against the PD-1/PD-L1 interaction was evaluated using a cell-based luminescence reporter assay. Briefly, PD-L1/T cell receptor (TCR) activator CHO (Chinese hamster ovary) cells were used as stimulator cells, and PD-1/NFAT-Luc Jurkat cells were used as reporter effector cells. The assay is based on the suppression of TCR/NFAT-driven luciferase activity by PD-1/PD-L1 engagement. Blockade of the PD-1/PD-L1 interaction restores NFAT-dependent luciferase expression, resulting in an increased luminescence signal.

PD-L1/TCR activator CHO cells were seeded into white opaque 6-well plates at 20,000 cells per well in RPMI 1640 and allowed to attach for 24 h. PD-1/NFAT-Luc Jurkat cells were then added at 20,000 cells per well. D-peptides PBDP-1 and PBDP-2 were diluted in assay medium and added to the coculture at the indicated concentrations. Nivolumab was included as a positive-control PD-1/PD-L1 blocking antibody, and vehicle-treated cocultures were used as negative controls. Additional control wells included Jurkat cells alone and Jurkat cells cocultured with CHO cells without inhibitor.

After incubation at 37 °C with 5% CO₂ for 6 h, the plates were equilibrated to room temperature for 15 min. Luciferase substrate was added to each well according to the manufacturer’s instructions, and luminescence was measured using a microplate reader. Relative luciferase activity was calculated by normalizing the luminescence signal to the indicated control group. Data were obtained from 3 independent replicates. Statistical significance was analyzed using unpaired *t* test, and *P* < 0.05 was considered statistically significant.

## Data Availability

The code of Mirror-Peptidizer is available at https://github.com/dahuilangda/Mirror-Peptidizer. Research data supporting this publication are available upon request.
