# Supplementary material for: Mirror-Peptidizer: In Silico Mirror-Image Screening Enables De Novo Design of D-Peptide Binders without D-Protein Synthesis
Source: Research (Wash D C). 2026 Aug 31;9:1420. doi: 10.34133/research.1420 (PMC13527210; doi:10.34133/research.1420)
Supplement: Supplementary file 1 [file research.1420.f1.zip › Supplementary Information revised 0809.docx]

Supplementary Information

for

**Mirror-Peptidizer: In Silico Mirror-Image Screening Enables De Novo Design of D-Peptide Binders without D-Protein Synthesis**

Supplementary Figures

Figure S1

Figure S1. Detailed structural analysis of MBDP2 (A), and MBDP3 (B) binding to MDM2 protein.

Figure S2

Figure S2. HPLC and MS analysis of MBDP 1-9.

Figure S3

Figure S3. NMR analysis of MBDP-1 and MDM2 protein. A. Overlay of ^1^H-^15^N HSQC spectra of MDM2, obtained during the automated titration experiment with MBDP-1. B. Histogram of maximum chemical shift perturbations (Δδmax) plotted as a function of the amino acid sequence of MDM2.

Figure S4

Figure S4. Raw ITC data between MBDP1-4 to MDM2 protein.

Figure S5

Figure S5. ITC analysis of the binding affinity of MBDP 6-7 to MDM2 protein.

Figure S6

Figure S6. ITC analysis of the binding affinity of MBDP1 with mutation MDM2 proteins.

Figure S7

Figure S7. The correlation between the binding potency of MBDPs with the MPNN scores.

Figure S8

Figure S8. Bio-layer interferometry (BLI) analysis of the interaction between MBDP-1 and MDM2. Representative BLI sensorgrams show concentration-dependent binding of MBDP-1 to recombinant human MDM2 at concentrations of 4.1-1000 nM (left). Steady-state analysis of the equilibrium responses yielded a K*_D_* of 29 nM (right).

Figure S9

Figure S9. HPLC and MS analysis of PBDP 1-4.

Figure S10

Figure S10. Raw ITC data between PBDP1-2 to PD-L1 protein.

Figure S11

Figure S11. ITC analysis of the binding affinity of PBDP 3-4 to PD-L1 protein.

Figure S12

Figure S12. ITC analysis of the binding affinity of PBDP1 with K75A mutation PD-L1 protein.

Figure S13

Figure S13. HPLC and MS analysis of IBDP-1.

Figure S14

Figure S14. Bio-layer interferometry analysis of the interaction between IBDP-1 and IL-23R. Representative BLI sensorgrams show concentration-dependent binding of IBDP-1 to recombinant human IL-23R at concentrations of 4.1-1000 nM (left). Steady-state analysis of the equilibrium responses yielded a K*_D_* of 490 nM (right).

Figure S15

Figure S15. Representative structural diversity of Chroma-generated peptide-target complexes. Representative peptide binders occupying distinct binding poses and/or adopting different backbone conformations are shown for (A) MDM2, (B) PD-L1, and (C) IL-23R. The corresponding D-peptide sequences are indicated below each structure.

Figure S16

Figure S16. D/L comparative benchmarking and stereochemical quality control of mirror-derived designs. (A) ProteinMPNN score distributions of direct L-peptide designs and mirror-derived D-peptide designs for PD-L1, MDM2, and IL-23R. Dashed lines indicate median values. KS statistics and p values are shown in each panel. (B) t-SNE visualization of the top-100 candidates from direct L-peptide and mirror-derived D-peptide design. Each point represents one candidate sequence. (C) Representative structural comparison of direct L-peptide candidates and mirror-derived D-peptide candidates. For visualization, mirror-derived complexes were transformed back into the native L-target coordinate frame. The two design modes show different binding poses, backbone trajectories, and secondary-structure arrangements. (D) Backbone dihedral-angle quality control of direct L-peptide and mirror-derived D-peptide designs. Ramachandran φ/ψ distributions show the expected mirror-related relationship between L- and D-backbones, supporting stereochemical consistency after coordinate mirroring.

Figure S17

Figure S17. Module-level ablation analysis of Mirror-Peptidizer. (A) Best ProteinMPNN scores from three workflows on PD-L1, MDM2, and IL-23R. Lower scores indicate better sequence–backbone compatibility. (B) Best synthesis penalties of the corresponding top candidates. Lower values indicate lower predicted synthesis risk. Chroma+ProteinMPNN favored structural compatibility, Chroma+BO favored synthetic tractability, and the complete workflow balanced both objectives.

Supplemental tables

Table S1. D-peptide design funnel.

| **Stage** | **MDM2** | **PD-L1** | **IL-23R** |
| --- | --- | --- | --- |
| Peptide lengths scanned | 7–19 aa | 8–20 aa | 12–28 aa |
| Chroma backbones generated | 400 | 400 | 399 |
| Backbones passing surface-geometry QC | 225 | 194 | 158 |
| ProteinMPNN sequences generated (10/backbone) | 4,000 | 4,000 | 3,990 |
| Sequences retained after geometry + complexity filtering | 2,007 | 1,808 | 1,266 |
| Unique sequences retained | 1,804 | 1,676 | 1,262 |
| Sequence variants explored by Bayesian optimization | 1,026 | 1,032 | 1,110 |
| Candidates prioritized for synthesis | MBDP series | PBDP series | IBDP series |

Table S2. ProteinMPNN configuration.

| **Item** | **Value** |
| --- | --- |
| Software / checkpoint | ProteinMPNN (dauparas/ProteinMPNN), vanilla v_48_020 (48 edges, 0.20 Å noise) |
| Network | hidden 128; 3 encoder + 3 decoder layers; backbone noise off at inference |
| Input preprocessing | parse_PDB → StructureDatasetPDB(max_length=10000); no fixed positions, omit, tie, PSSM or bias |
| Design / fixed chains | binder chain designed; all receptor chains fixed |
| Sampling | temperature 0.1; 8–10 sequences/backbone |
| Score | mean NLL over designed-chain positions; lower = more backbone-compatible |
| Command | python run_design.py --receptor <target.pdb> --len_binder L --temperature 0.1 --num_poses N --num_seqs_per_pose 10 |

Table S3. Bayesian-optimization configuration.

| **Item** | **Value** |
| --- | --- |
| Surrogate | Gaussian process, Matérn-5/2 kernel, ARD length scales, log-marginal-likelihood fit (120 iters) |
| Encoding | one-hot (L×20) |
| Acquisition | UCB (β = 0.2) |
| Explorer | BO_EVO (1–3 substitutions/step; batch min Hamming 2) |
| Initial observations | the pose’s ProteinMPNN candidates (8–10) |
| Iterations | 8 rounds × 10 candidates (5 rounds in the ablation study) |
| Fitness | −MPNN_score − 0.5 × synthesis_penalty |
| Synthesis-risk term | SPPS coupling-step difficulty + aggregation propensity + longest difficult-coupling run |
| Stopping | fixed rounds (no early stopping) |

Table S4. Core metrics of three ablation workflows on PD-L1, MDM2, and IL-23R.

| Target | Mode | *n* | Best score | Best penalty | Best risk |
| --- | --- | --- | --- | --- | --- |
| PDL1 | Chroma+ProteinMPNN | 3670 | **1.298** | 2.159 | high |
| PDL1 | Chroma+BO | 1880 | 2.463 | **0.112** | low |
| PDL1 | Chroma+ProteinMPNN+BO | 1841 | 1.758 | 0.135 | low |
| MDM2 | Chroma+ProteinMPNN | 3880 | **0.992** | 1.146 | moderate |
| MDM2 | Chroma+BO | 2280 | 2.340 | 0.188 | low |
| MDM2 | Chroma+ProteinMPNN+BO | 2207 | 1.594 | **0.150** | low |
| IL23R | Chroma+ProteinMPNN | 3590 | **1.349** | 1.252 | moderate |
| IL23R | Chroma+BO | 2700 | 2.574 | **0.139** | low |
| IL23R | Chroma+ProteinMPNN+BO | 2671 | 1.676 | 0.193 | low |

Table S5. Summary of Tier-1 D-peptide candidate generation for additional targets.

| Target | PDB | *n* | Unique seq. | Best score | Mean score | Top‑100 mean |
| --- | --- | --- | --- | --- | --- | --- |
| TNF‑*α* | 1TNF | 340 | 273 | 2.0915 | 2.4764 | 2.2764 |
| CXCR2 | 8XXH | 1,757 | 1,571 | 1.4584 | 2.0021 | 1.6745 |
| CXCR4 | 8K3Z | 1,911 | 1,796 | 1.3946 | 1.9574 | 1.5681 |
